# Genomic tagging of endogenous Type IIβ Phosphatidylinositol 5-phosphate 4-kinase in DT40 cells reveals a nuclear localisation

**DOI:** 10.1016/j.cellsig.2007.01.010

**Published:** 2007-06

**Authors:** Jonathan P. Richardson, Minchuan Wang, Jonathan H. Clarke, Ketan J. Patel, R.F. Irvine

**Affiliations:** a Department of Pharmacology, University of Cambridge, Tennis Court Road, Cambridge CB2 1PD, UK; b Laboratory of Molecular Biology, Hills Road, Cambridge CB2 2QH, UK

**Keywords:** Type II PIPkin, Type II Phosphatidylinositol 5-phosphate 4-kinase, Phosphatidylinositol 5-phosphate, Phosphatidylinositol 5-phosphate 4-kinase, DT40 cells, Genomic tagging

## Abstract

Previous studies from acutely transfected HeLa cells have identified an acidic α-helix in the Type IIβ PtdIns5*P* 4-kinase (PIPkin IIβ) as a putative novel nuclear localisation sequence (Ciruela et al. Biochem. J. 364, 587–591 2000). However, some heterogeneity in cellular localisation was always observed, and other published aspects of PIPkin IIβ physiology are more consistent with an extra-nuclear function. As a means of resolving whether the endogenous PIPkin IIβ is nuclear, we have used the high homologous recombination frequency of DT40 cells to knock an epitope tag (Mosedale et al., Nat Struct Biol. 12, 763–771 2005) into one of the alleles of the DT40 PIPkin IIβ gene. We show that PIPkin IIβ is expressed as a tagged protein, is active as revealed by immunoprecipitation and enzyme assay, and that cellular fractionation reveals that it is indeed nuclear. Genomic tagging of endogenous proteins in DT40 cells is a technique that offers unique advantages in studying endogenous signalling proteins.

## 1 Introduction

Inositol lipids have proliferated in their number and functions over the last twenty years such that they are now known to participate in many cellular processes. Although most of these functions are in the cytoplasm, there are also well defined functions within the nucleus (for reviews see [1–5]). In particular, the function of PtdIns(4,5)*P*_2_ in the nucleus has been a major focus of study, both in its own right as a lipid regulator of proteins, and as a precursor for diacylglycerol to activate nuclear PKC [1,4,5]. Thus the synthesis of PtdIns(4,5)*P*_2_ in the nucleus has been extensively studied, but is not yet fully understood.

Radiolabelling experiments on isolated nuclei suggest that the major route of synthesis of PtdIns(4,5)*P*_2_ is by the expected 5-phosphorylation of PtdIns4*P* [6]. The nuclear synthesis of PtdIns4*P* from PtdIns is probably catalysed by the Type IIIβ PtdIns 4-kinase, which has been shown to be partly nuclear in its localisation [7,8], and then the synthesis of PtdIns(4,5)*P*_2_ must proceed by a Type I PtdIns*P* kinase (PIPkin), most likely the Type Iα [4,9] (human nomenclature — the mouse homologue is PIPkin Iβ). However, there is also evidence in the nucleus for the alternative route of PtdIns(4,5)*P*_2_ synthesis [10], which is the 4-phosphorylation of PtdIns5*P* by Type II PIPkins. For example, PtdIns5*P* is found in the nucleus and its nuclear levels change with the cell cycle [11] or when cells are stressed [12], though its route of synthesis is currently unknown.

**Fig. 1:**
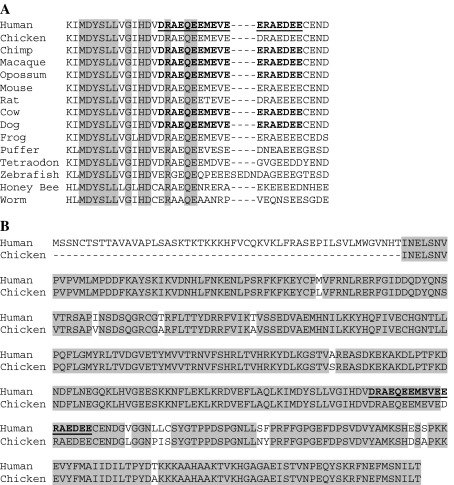
Nuclear localisation sequences in Type IIβ PIPkins. A. This is a line-up of residues 275 to 310 of human Type IIβ PIPkin and the corresponding residues of other species, with the human nuclear localisation sequence described by Ciruela et al. [13] underlined; species with an identical localisation sequence are in bold. B. The complete sequence of the human enzyme is shown, with the chicken sequence aligned with it (note that the start of the chicken enzyme is unclear from the database). The human nuclear localisation sequence is underlined.

Of the Type II PIPkins, PIPkin IIβ has also been suggested to be primarily nuclear, and it may be that a fraction of PIPkin IIα is nuclear also [9,13]. We previously showed that the nuclear localisation of acutely transfected PIPkin IIβ was absolutely dependent on a novel nuclear localisation sequence [13] consisting of an acidic α-helix 16 amino acids long, numbered α-helix 7 in the PIPkin IIβ structure described by Rao et al. [14]. We also showed more recently that mutating a single amino acid within that α-helix, substituting Met 296 for a Thr, caused a significant, though not complete, shift to a cytoplasmic distribution [15]. A line-up of Type II PIPkins shows that this α-helix is highly conserved in vertebrates (Fig. 1A) (though interestingly the rat sequence has the same 296 Met–Thr subsitution), with the exception of Zebrafish, where there is an insertion of four extra amino acids. From our earlier work [13] we can infer that in invertebrates (Fig. 1) and possibly in *Danio*, PIPkin IIβ will not be nuclear, and in rat it will be only partly nuclear [15]. Recently, Gozani et al. [16] have presented evidence that transfected PIPkin IIβ can modulate PtdIns5*P*-mediated events in the nucleus, and Jones et al. have implicated phosphorylation by p38 stress kinase in the regulation of nuclear PIPkin IIβ [12].

However, set against all this evidence for a nuclear localisation and function for PIPkin IIβ is the fact that it was first cloned by its apparent interaction with a cell surface receptor — that for TNF-α [17]. Moreover, PIPkin IIβ has subsequently been reported to associate with the EGF receptor [18]. The knock-out mouse for PIPkin IIβ shows a phenotype of increased insulin sensitivity [19], consistent with the observations from Carricabura et al. [20], who showed that increasing PIPkin IIβ by transfection resulted in decreased PtdIns(3,4,5)*P*_3_ production in response to insulin, probably caused by increased PtdIns(3,4,5)*P*_3_ degradation. The suggested mechanism was that PtdIns5*P* is a stimulator of PtdIns(3,4,5)*P*_3_ phophatase(s) [20]. All these data together suggest a (perhaps major) cytoplasmic function for PIPkin IIβ. Finally, in all our transfection experiments, we frequently saw some cytoplasmic PIPkin IIβ present, even when it was mostly nuclear, and indeed, there were also 5–10% of cells in every transfected dish with very little in the nucleus and the majority in the cytoplasm [13]. Moreover, differences between transfected and endogenous localisations have also been observed with Type I PIPkins (e.g. human Type Iβ PIPkin [21,22]).

Thus we are in a position with PIPkin IIβ, as with many other proteins, that acute transfection may not be giving the full picture of its physiological location and regulation — indeed we actually have no evidence for a nuclear location of the endogenous enzyme. One way round this problem in the absence of a highly isoform-specific antibody is to tag the endogenous protein genetically. This can be done comparatively easily by exploiting the extremely high rate of homologous recombination of DT40 cells [23], as exemplified by the tagging of Hef, a protein component of the Fanconi anemia-related tumor-suppressor complex, by Mosedale et al. [24]. Here we describe the generation and characterisation of a DT40 cell that expresses active PIPkin IIβ tagged at its C-terminus. We use this cell line to show that PIPkin IIβ is indeed a nuclear enzyme.

## 2 Materials and methods

### 2.1 Cell culture, transfection and visualisation

DT40 cells were cultured in RPMI 1640 plus glutamine supplemented with 7% (v/v) foetal calf serum, 3% (v/v) heat-treated chicken serum, 1% (v/v) penicillin/streptomycin solution (GIBCO) and 5 mM 2-mercaptoethanol at 37 °C, 5% CO_2_. Puromycin was added at a final concentration of 500 ng/ml where appropriate. For transfection, 20 × 10^6^ cells were harvested, washed and resuspended in 600 μl of cold PBS and mixed with 30 μg of linear homologous recombination cassette DNA (for molecular tagging of the type IIβ PIPkin) and transferred to a chilled electroporation cuvette. Transfection was by the Nucleofector method (Amaxa Biosystems). Cells were placed on ice for 5 min, diluted in DT40 medium and allowed to recover for 24 h at 37 °C, 5% CO_2_. Potential homologous recombinants were transferred to 96 well plates and selected by the addition of puromycin. Resistant colonies were observed after 9 days and were cultured for further analysis. For GFP studies (acute transfection), 10 μg of plasmid DNA was similarly transfected and cells allowed to recover for 24 h at 37 °C, 5% CO_2_ in dishes containing DT40 medium and poly-d-lysine coated cover slips. After recovery, cover slips were washed 3 times in sterile PBS and cells fixed for 45 min on ice with 0.1 M NaH_2_PO_4_ pH 7.4 containing 4% (v/v) paraformaldehyde. Cover slips were washed in PBS and mounted on microscope slides with 20 μl ProLong Gold Antifade reagent (Molecular Probes). Cells expressing GFP-tagged PIPKIN were visualised with a Zeiss Axiovert 100 M confocal microscope.

### 2.2 Molecular tagging of Type IIβ PIPkin

**Fig. 2:**
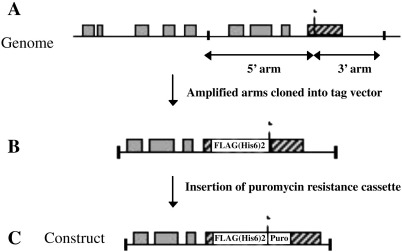
Schematic depiction of strategy for making constructs for genomic tagging in DT40 cells. See Methods section and text for further details.

A summarised depiction of the strategy used is shown in Fig. 2. The sequence of *Gallus gallus* (DT40) genomic DNA was obtained from the ensembl genome browser (http://www.ensembl.org). To generate flanking regions of homology required to tag the type II Beta PIPkin, primer pairs designed to facilitate directional cloning were used to PCR amplify regions immediately 5′ and 3′ of the type II beta PIPkin stop codon (2.2 and 3.6 kb respectively), from DT40 genomic DNA using the LA-PCR kit (TAKARA BIO, Japan). The 5′ arm was amplified with the forward primer: (5′-CATATCGATGTGTGCAGTTTGTCTCAGTCC-3′) and reverse: (5′-TACTCTAGATGTCAGGATATTGGACATAAATTC-3′). The 3′ arm was amplified with forward: (5′-CATGGATCCTCATCTCCAGCCTTCAGAGG-3′) and reverse: (5′-TACGCGGCCGCAGAGCAAGGAGAATCAGTAGTG-3′). Amplified arms were sub cloned separately into pBluescript SK+ (Stratagene) and confirmed by DNA sequencing. Arms were then individually sub cloned into a pBluescript derivative containing an XbaI–FLAG–(His6)_2_–STOP sequence immediately followed by a unique BamHI restriction site. A puromycin drug resistance cassette was then cloned into the BamHI site (Fig. 2). DNA was prepared by endotoxin free maxi-prep (Qiagen) and linearised prior to transfection. Integration of the tag at the correct genomic location was confirmed by PCR analysis of genomic DNA from transfected, puromycin resistant cells using primers internal [5′-GGAGAGTGAAGCAGAACGTGG-3′] and external [5′-CCTCAGCTCCGACGTTGCCATG-3′] to the site of genetic recombination.

### 2.3 Extraction of genomic DNA

15 × 10^6^ cells were harvested, washed in sterile PBS and resuspended in 0.5 ml of (100 mM Tris–HCl pH 8.5, 5 mM EDTA pH 8.0, 0.2% SDS, 200 mM NaCl, 100 μg/ml proteinase K). Cells were incubated at 37 °C for 4 h and genomic DNA precipitated by addition of an equal volume of isopropanol. Genomic DNA was pelleted by centrifugation at 13,000 rpm for 5 min and washed once in 70% ethanol. Pellets were resuspended in 100 μl of 10 mM Tris–HCl pH 8.0 containing 60 μg of RNase A and incubated at 37 °C until dissolved.

### 2.4 Preparation of protein extracts

50 × 10^6^ cells were harvested, washed in PBS once, and resuspended in protein extraction buffer (1 × PBS containing 5 mM EDTA pH 8.0, 1% (v/v) triton X-100, 1 mM PMSF and 1:10 (v/v) of protease inhibitor cocktail (Sigma)). Cells were lysed on ice water for 20 min and the lysate cleared by centrifugation at 13,000 rpm for 5 min at 4 °C. Protein concentrations were estimated with the detergent compatible protein assay kit (Biorad).

### 2.5 Fractionation of cells

DT40 WT and JPR3 cells (a clone with Type IIβ PIPkin tagged — see Results) were grown to approximate 2.5 × 10^6^ cells/ml in medium. 400 ml of each culture was harvested and washed in PBS once. Cell pellets were resuspended in 0.813 ml of 1× swell buffer (5 mM Tris–HCl pH 7.4 with 1.5 mM KCl and 2.5 mM MgCl_2_) in 50 ml tubes. Tubes were placed on ice for exactly 10 min. 33 μl of cold 33 mM EGTA (pH7.4) was added and then cells were syringed through a 23-gauge needle ten times. Immediately after syringing, 160 μl of sucrose solution (1.8 M sucrose prepared in lysis buffer (10 mM Tris–HCl pH 7.4 with 1 mM EGTA, 1.5 mM KCl and 5 mM MgCl_2_)) followed by 2.7 μl of 1 M MgCl_2_ solution were added.

Samples were layered onto 4 ml of 2.3 M sucrose (prepared in lysis buffer) and spun at 35,000 rpm for 1 h on a SW55Ti rotor using Beckman Optima L-100 XP Ultracentrifuge. After spinning, a 300 μl volume was collected from the top of the gradient, which we took to be representative of the cytoplasmic fraction. The nuclei (white pellet on the bottom of the centrifuge tube) were resuspended in 1 ml of 0.32 M sucrose in lysis buffer. Nuclei were pelleted, and the absence of contaminating whole cells was confirmed by microscope examination. They were lysed in 75 μl of lysis buffer P1 (containing 1 × PBS, 0.1% (v/v) Triton X-100 and 0.5% protease inhibitor cocktail) on ice for 20 min. Samples of both cytoplasmic and nuclear fractions were taken for SDS PAGE and Western blotting (below) with anti-histone or anti-actin antibodies (to assess purity), and the remainder was used for affinity purification of FLAG–His6 tagged proteins using TALON beads as described below.

### 2.6 SDS PAGE and Western blotting

Protein samples were resolved by electrophoresis on 12.5% polyacrylamide gels using the Protean III system (Biorad). Electrophoresed proteins were transferred to nitrocellulose membrane using a Trans-blot transfer cell (Biorad). Membranes were blocked in 1 × PBS containing 0.1% (v/v) tween-20 and 4% (w/v) fat free milk powder and probed with the appropriate antibodies diluted in the same solution. Unbound antibodies were washed off with 1 × PBS containing 0.1% (v/v) tween-20, and after addition of HRP-linked secondary antibodies, the blot was incubated with Super Signal Western blot development reagent (Pierce). Protein bands were quantified by densitometry using a GeneGnome 50000 (Syngene, MD, USA), and then visualised for Figures by exposure to film. Antibodies used were: Anti-β-actin monoclonal antibody (AC74, Sigma); Anti-histone H3 polyclonal antibody (ab1791, Abcam); Anti-FLAG M2 monoclonal antibody (200472, Stratagene).

### 2.7 Affinity purification of FLAG–His6-tagged proteins

Cells were harvested, washed in PBS and lysed in protein extraction buffer as described. Typically, 50 μl of Talon metal affinity resin (Invitrogen) was pre washed 3 times in 1 ml of protein extraction buffer and added to 0.8 mg of whole cell protein extract made up to a total volume of 250 μl with protein extraction buffer. Samples were incubated on a rotating wheel for 2 h at 4 °C. Resin was pelleted by centrifugation at 10,000 rpm and washed 3 times in wash buffer (50 mM sodium phosphate buffer pH 7.0, 300 mM NaCl, 1% (v/v) glycerol, 1:200 (v/v) protease inhibitor cocktail) and bound proteins were eluted in 50 μl of elution buffer (50 mM sodium phosphate buffer pH 7.0, 300 mM NaCl, 1% (v/v) glycerol, 150 mM imidazole, 1:200 (v/v) protease inhibitor cocktail). Eluted proteins were submitted to SDS PAGE as described above, blotted with anti-FLAG antibodies.

### 2.8 Immunoprecipitation of FLAG–His6-tagged proteins

750 × 10^6^ cells were harvested, washed in PBS and lysed in 2 ml of protein extraction buffer as described. Clearing the crude lysate by centrifugation yielded approximately 1.8 ml of protein extract, to which 3 μg of anti His6 monoclonal antibody (B D Biosciences) was added. Antibody containing extracts were incubated on ice for 1 h with occasional mixing by inversion. Protein G sepharose was equilibrated by washing 3 times in 1 ml of protein extraction buffer and 50 μl added to samples which were incubated on a rotating wheel at 4 °C for 16 h. Sepharose was pelleted by centrifugation at 2000 rpm and washed twice in 1 ml of cold TBS and once in 1 ml of cold PBS. Immunoprecipitated proteins were prepared for kinase activity assay by washing the pellet in 500 μl of PIPkin reaction buffer (50 mM Tris–HCl pH 7.4, 80 mM KCl, 10 mM Mg acetate, 2 mM EGTA).

### 2.9 Kinase activity assay

Immunoprecipitated proteins were resuspended in 50 μl of 10 mM Tris–HCl pH 7.4 and 100 μl of 2× PIPkin reaction buffer and combined with substrate (60 μM phosphatidylethanolamine, 6 μM phosphatidylinositol-5-phosphate sonicated in 50 μl of 10 mM Tris–HCl pH 7.4 to form micelles). Kinase reactions were initiated by the addition of 5 μCi of γ–^32^P–ATP (New England Nuclear, Boston USA) and incubated at 30 °C for 2 h. Reactions were stopped by addition of 500 μl CHCl_3_:CH_3_OH (1:1 v/v) and 125 μl 2.4 N HCl. Folch lipids [25] (20 μl) extracted from porcine brain were added as carrier. Phosphorylated lipids were extracted as described [11] and resolved by thin layer chromatography on silica-coated glass plates (activated by exposure to 50% (v/v) CH_3_OH, 1% (w/v) potassium oxalate, 2 mM EGTA followed by baking at 110 °C for 1 h) in a pre-equilibrated Whatman 3MM paper-lined tank. TLC plates were developed in solvent phase (28:40:10:6 (v/v) CHCl_3_:CH_3_OH:H_2_O:NH_4_OH) until solvent front reached to the top of the plate. Radioactive lipids were visualised by autoradiography.

### 2.10 Site-directed mutagenesis

Site-directed mutagenesis was performed on the human Type IIβ PIPkin cloned into pEGFP–C1 (Clontech) using the QuikChange mutagenesis method (Stratagene), to introduce an Glu300Asp amino acid substitution into the human nuclear localisation sequence producing the equivalent chicken sequence: (289-DRAEQEEMEVE[E300D]RAEDEE-306). Mutagenesis was confirmed by DNA sequencing.

## 3 Results and discussion

### 3.1 Localisation of acutely transfected PIPkins in DT40 cells

**Fig. 3:**
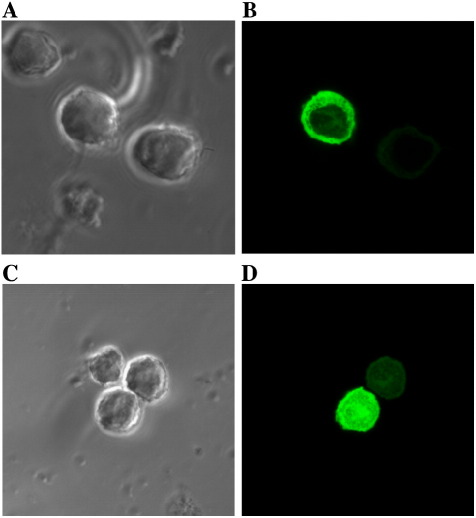
Localisation of human Type II PIPkins acutely transfected into DT40 cells. DT40 cells were acutely transfected with GFP-tagged Type II PIPkins, and imaged with light microscopy (A and C) or by confocal flourescence microscopy (B and D). A and B, Type IIα PIPkin; C and D, Type IIβ PIPkin with a Glu300Asp mutation (see text and Fig. 2C).

The chicken sequence of PIPkin IIβ is shown in Fig. 1B, and reveals that there is an Aspartate instead of Glutamate at residue 300 in the relevant α-helical nuclear localisation sequence [11]. This is unlikely to alter the structure of the α-helix, as determined by sequence analysis [13,26], but nevertheless we mutated that residue in our GFP-tagged human clone to Aspartate before comparing the localisation of the human PIPkin IIα and IIβ in DT40 cells. As we found previously, they were respectively predominantly cytosolic and nuclear (Fig. 3), although the degree to which PIPkin IIβ is cytosolic is difficult to assess from these images. However, the difference between them is very similar to that observed in HeLa cells [11], confirming that the nuclear transport mechanism that recognises this localisation sequence is functional in acutely transfected DT40 cells.

### 3.2 Genomic tagging

Fig. 2 shows in outline the strategy used in tagging PIPkin IIβ in DT40 cells (see Materials and Methods, and Ref. [24] for more details). In brief, the last 2.6 kbp of the PIPkin IIβ gene, and the following 3.6 kbp base pairs, were cloned and inserted into a plasmid which placed a FLAG–(His6)_2_ double-tag, followed by a puromycin selection cassette between them. Transfection of this into DT40 cells followed by puromycin selection produces cells which have the tag attached to the C-terminus of the protein [24] when expressed by one of the two alleles. All our experiments were conducted on one clonal line derived from this procedure, which we called JPR3.

**Fig. 4:**
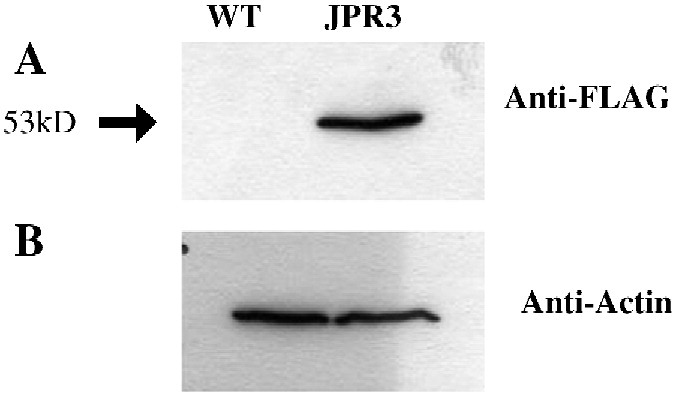
Nickel bead pull-down of FLAG–(His6)_2_–tagged PIPkin IIβ from DT40 cells. Wild type cells (left) or PIPkin IIβ-tagged cells (JPR3) were lysed, and a nickel bead pull-down, imadazole elution and Western blotting with anti-FLAG antibody was performed as described in the Methods section (A). A portion of the lysate from each cell preparation was kept and blotted for actin to control for similar numbers of cells used (B). The expected position of a 53 kD MW protein (the same as FLAG–(His6)_2_ tagged PIPkin IIβ) is indicated by the arrow on Fig. 4A.

**Fig. 5:**
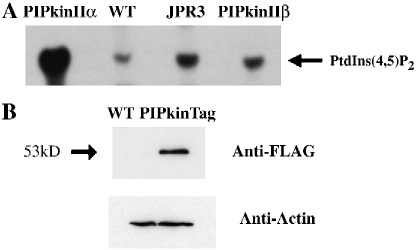
Activity of tagged PIPkin IIβ in DT40 cells. Wild type cells (WT) or cells with PIPkin IIβ tagged with FLAG–(His6)_2_ (JPR3) were lysed and split into two portions. A. One half was immunoprecipitated with anti-FLAG antibody, and the precipitate assayed for PtdIns5*P* 4-kinase activity, as described in the Methods section. Also assayed were recombinant (human) PIPkin IIα and PIPkin IIβ enzymes (these two at the same protein concentration). The location of the radioactive PtdIns(4,5)*P*_2_ spot is indicated with an arrow. Spots were quantified by densitometry or by scraping and scintillation counting. B. The other half of each lysate was processed by a nickel bead pull-down, imidazole elution and Western blotting with anti-FLAG, to confirm expression of Type IIβ PIPkin. The expected position of a 53 kD MW protein (the same as FLAG–(His6)_2_ tagged PIPkin IIβ) is indicated by the arrow on Fig. 5B.

Successful integration of the tag at the correct genomic location was confirmed by PCR analysis of the corresponding genomic DNA (see Methods), and the expression of the tagged protein was demonstrated by the experiment shown in Fig. 4. A Nickel bead pull-down followed by blotting with an anti-FLAG antibody revealed a single clear band (of the correct molecular weight to be PIPkin IIβ), which was absent in Wild-type cells. To confirm that this is tagged PIPkin IIβ and is correctly folded, we immunoprecipitated it from the tagged cells with anti-His6 antibody and assayed for PtdIns5*P* 4-kinase activity, with recombinant Human PIPkins IIα and IIβ as controls. As we have found before, PIPkin IIβ is much less active than PIPkin IIα under these assay conditions (Fig. 5), but nevertheless from the ‘tagged’ cells we were able to immunoprecipitate activity that was significantly above the background from Wild-type cells. In three independent experiments, the ratio (‘tagged’ cells to Wild-type) of immunoprecipitated enzyme activity, quantified by densitometry or by scintillation counting, was 2.37 ± 0.27 (this is different from unity with a *P* < 0.01 by Student's *t* test).

### 3.3 Localisation of PIPkin IIβ

The level of expression of PIPkin IIβ was too low for immunocytochemistry using either anti-FLAG or anti-His antibodies (we found that DT40 cells had a high background with both sets of antibodies — significantly higher than several mammalian cell lines in comparative experiments). However, we could gain quantitative insight into its localisation by fractionating DT40 cells into nuclear and cytoplasmic fractions, blotting a portion of these with anti-actin and anti-H3 histone as markers for the purity of the fractions, and then quantifying the PIPkin IIβ content of the remainder by Nickel bead pull-down followed by anti-FLAG blotting, as described above (Fig. 4).

**Fig. 6:**
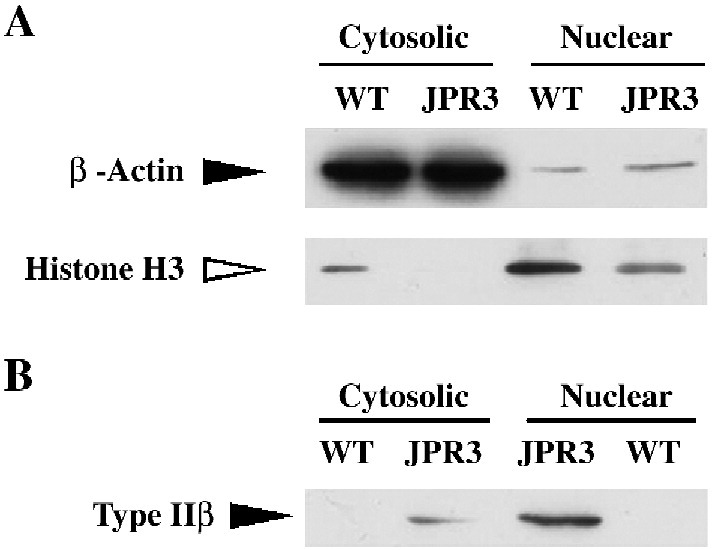
Nuclear and cytoplasmic fractions of DT40 cells. Wild type (WT) or cells with PIPkin IIβ tagged with FLAG–(His6)_2_ (JPR3) were fractionated into cytoplasmic and nuclear fractions as described in the Methods section. A portion of each fraction was Western-blotted for Histone H3 or actin, and the rest was submitted to the standard nickel bead pull-down followed by imidazole elution and Western blotting with anti-FLAG (see Fig. 4 and Methods), to quantify the amount of tagged PIPkin IIβ.

We assessed the cytoplasmic contamination of the nuclear fractions by blotting for β-actin. This is not a perfect marker, as we would expect a small percentage of actin to be truly nuclear (see [1,27]), but it served to confirm independently our microscopical examinations, which told us that the cells had been lysed and that we had prepared pure nuclei. The predominance of PIPkin IIβ in the nuclear fraction demonstrates the nuclear localisation of the endogenous enzyme. A key question is: how much of the small amount of PIPkin IIβ in the cytoplasmic fraction (Fig. 5B) can be accounted for by nuclear disintegration or by leakage of PIPkin IIβ during the fractionation? We have used a histone antibody as a marker for this, but there is no perfect marker in this context. Histone H3 has a lower molecular weight than PIPkin IIβ, so it could be argued to be more likely to leak out than the PIPkin; however, Histone H3 is also integral to chromatin structure whereas PIPkin IIβ is probably not, and so Histone H3 could be argued to be less likely to leak out. As we do not know to what degree PIPkin IIβ is freely diffusable in the nucleoplasm, these arguments limit the degree to which we can unambiguously assign a complete nuclear localisation to PIPkin IIβ, and they also make it pointless to pursue other nuclear markers - each will suffer from similar *caveats*. However, the data in Fig. 6 do enable us to say that within the error limits of our protocol, the endogenous PIPkin IIβ in these cells is mostly nuclear.

In conclusion, we have shown that genomic tagging of PIPkin IIβ reinforces our earlier acute transfection studies [13]. The issue of whether there is any cytoplasmic PIPkin IIβ at all, as some experiments imply (see Introduction), cannot be resolved by our data. But we can support the likely physiological significance of nuclear PIPkin IIβ and PtdIns5*P* [11–13,16], and importantly, our data also confirm the reality and novelty of PIPkin IIβ's unusual nuclear localisation sequence [13]. Finally, we suggest that this technique may be useful to study other signalling molecules for which transfection or a lack of good antibodies is a hindrance. As well as exploring the true localisation of a protein (as we have done here), this approach enables its physiological interactions with other proteins, or changes in localisation or post-translational modifications to be followed in a clear and unambiguous way.
